# Isolated Flaccid Limb Weakness in a Toddler: The Timing of Spinal MRI Matters

**DOI:** 10.7759/cureus.110640

**Published:** 2026-06-11

**Authors:** Young Hwan Kim, Kye Hyang Lee

**Affiliations:** 1 Department of Pediatrics, Daegu Catholic University School of Medicine, Daegu, KOR

**Keywords:** acute flaccid myelitis, flaccid limb weakness, guillain-barré syndrome, pediatric, spinal mri, ventral nerve root enhancement

## Abstract

Acute flaccid myelitis (AFM) and Guillain-Barré syndrome (GBS) share overlapping clinical features, making differentiation challenging. Although characteristic spinal cord gray matter lesions are typically present in AFM, magnetic resonance imaging (MRI) findings may evolve during the disease course. In later stages, isolated ventral nerve root enhancement can be observed without visible gray matter lesions, mimicking the radiologic features of GBS. We report a child in whom AFM was ultimately favored, although the initial radiologic impression suggested GBS. A 23-month-old boy developed acute flaccid weakness of the left leg shortly after a febrile illness, which persisted for one month before presentation. Spinal MRI revealed ventral nerve root enhancement without gray matter lesions, a pattern suggestive of GBS; however, the clinical presentation was more consistent with AFM. Over two years of follow-up, persistent unilateral weakness and marked limb atrophy supported AFM as the more likely diagnosis. This case highlights the temporal evolution of imaging findings in AFM and emphasizes that delayed imaging may obscure characteristic features, potentially leading to diagnostic confusion with GBS.

## Introduction

Acute flaccid limb weakness in children has a broad differential diagnosis, with Guillain-Barré syndrome (GBS) being the most common cause [1-3]. Acute flaccid myelitis (AFM) is a poliomyelitis-like syndrome characterized by acute flaccid paralysis (AFP) of one or more limbs associated with spinal cord gray matter lesions [2-4]. Following recent surges of AFP cases in the United States, AFM has become an essential diagnostic consideration in children presenting with acute flaccid weakness [1]. However, AFM and GBS often occur after a viral prodrome and may share overlapping clinical, electrophysiological, and radiologic features, making the distinction between them challenging [2,3]. The Centers for Disease Control and Prevention (CDC) case definition and recently proposed diagnostic criteria for AFM primarily rely on characteristic magnetic resonance imaging (MRI) abnormalities involving the spinal gray matter [2,5]. Importantly, MRI findings in AFM may evolve during the disease course. Gray matter lesions are typically observed during the acute phase but may become less conspicuous or resolve as the disease progresses, whereas ventral nerve root enhancement may emerge during the subacute stage [6,7]. Although quantitative data on stage-specific MRI sensitivity in AFM are limited, these temporal changes suggest that the diagnostic yield of spinal MRI depends on the timing of imaging. This timing-dependent evolution can complicate the distinction between AFM and GBS because nerve root enhancement is not specific to AFM and may also occur in GBS [3]. In particular, when spinal MRI is obtained beyond the acute phase, characteristic gray matter lesions may no longer be apparent, whereas isolated ventral nerve root enhancement may predominate and mimic GBS. Here, we report a child with isolated flaccid limb weakness in whom spinal MRI obtained one month after symptom onset demonstrated ventral nerve root enhancement as the sole abnormality. This case illustrates how MRI timing can contribute to diagnostic confusion between AFM and GBS. 

## Case presentation

**Figure 1:**
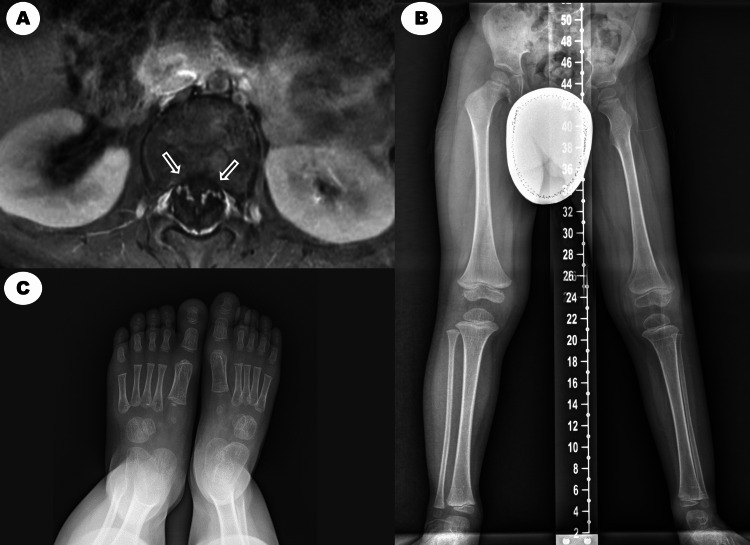
Spinal MRI and Follow-up Radiographs Demonstrating Chronic Left Lower Extremity Atrophy (A) Spinal MRI obtained one month after symptom onset demonstrates prominent enhancement of the bilateral ventral nerve roots. Arrows indicate the enhancing ventral nerve roots. (B) Radiograph obtained two years later shows diffuse atrophy of the left lower extremity extending from the hip to the foot. (C) A close-up radiograph of the left foot demonstrates distal atrophy compared with the contralateral side, consistent with chronic neurogenic atrophy.

A previously healthy 23-month-old boy presented with a one-month history of left leg weakness. He developed a fever of up to 39.3°C after a sick contact. Two days later, he suddenly had difficulty standing and walking and tended to fall to one side. The weakness was maximal at onset and persisted without substantial improvement despite extensive orthopedic evaluation. On examination, he was alert and calm, with no local signs of inflammation in the affected limb. Left leg strength was Medical Research Council grade 2, with movement possible only with gravity eliminated. The left leg was flaccid and areflexic, whereas the right leg was normal. Spinal MRI obtained on the first day of admission demonstrated prominent enhancement of the ventral nerve roots with mild smooth thickening of the cauda equina, which was interpreted as probable GBS (Figure 1A).

Electrophysiological studies showed markedly reduced compound muscle action potentials, preserved sensory nerve action potentials, and active denervation in the left lower extremity, suggesting a left lumbosacral root lesion or anterior horn cell involvement. Cerebrospinal fluid (CSF) analysis revealed a white blood cell count of 2/µL, a protein level of 38 mg/dL, and an IgG index of 0.69 (<0.85). Serum myelin oligodendrocyte glycoprotein IgG antibody and aquaporin-4 antibody tests were negative. CSF culture and multiplex meningitis polymerase chain reaction testing were also negative. Enterovirus was not detected in CSF, respiratory tract samples, or stool. Based on the clinical presentation and electrodiagnostic findings, AFM was favored as the leading diagnostic possibility, whereas GBS remained an alternative consideration given the radiologic findings. Intravenous immunoglobulin was administered for five days. During treatment, mild improvement in proximal muscle strength was observed; however, flaccidity and areflexia persisted. He was discharged with a plan for continued rehabilitation. By 10 months after onset, he was able to walk independently with a dragging gait, although marked atrophy of the left leg had developed. Two years after onset, he continued to walk independently with a limping gait; the left leg remained flaccid and areflexic, with diffuse atrophy consistent with chronic neurogenic changes (Figures 1B, 1C). The clinical timeline is summarized in Table 1.

**Table 1:** Timeline of Clinical Events CSF, cerebrospinal fluid; MRI, magnetic resonance imaging.

Time point	Clinical event
Day -2	Febrile illness after a sick contact
Day 0	Sudden onset of left leg weakness
One month after symptom onset	Admission, spinal MRI, CSF analysis, electrophysiological studies, and enterovirus testing
Hospital days 1-5	Intravenous immunoglobulin treatment
Ten months after symptom onset	Independent walking with a dragging gait and left leg atrophy
Two years after symptom onset	Persistent limping gait with flaccidity, areflexia, and diffuse left leg atrophy

## Discussion

AFM and GBS both commonly present with rapidly progressive limb weakness and reduced tendon reflexes after a prodromal illness. The diagnosis of AFM requires integration of clinical, laboratory, neuroimaging, and electrophysiological findings [2]. A recent comparative pediatric study found that AFM, compared with GBS, was more often associated with a shorter interval from symptom onset to nadir, asymmetric limb involvement, absence of sensory deficits, CSF pleocytosis, typical spinal cord lesions on MRI, and a more protracted course with persistent weakness [3]. In our patient, the clinical presentation one month after symptom onset was more consistent with AFM, whereas MRI findings raised concern for GBS. However, the absence of spinal gray matter lesions prevented him from meeting AFM diagnostic criteria, leaving the diagnosis equivocal at that stage. The asymmetric motor-predominant presentation, absence of sensory deficits, electrodiagnostic findings suggesting anterior horn cell or lumbosacral root involvement, and persistent unilateral weakness with neurogenic atrophy over more than two years collectively favored AFM over GBS. 

Spinal MRI is generally the most informative diagnostic tool for AFM. T2 hyperintensity of the spinal cord gray matter represents the radiologic hallmark of AFM and is a key component of current diagnostic criteria [2,5]. However, MRI findings may evolve during the disease course. In the early acute phase, lesions are often confluent and ill-defined, involving much of the gray matter with variable surrounding white matter edema. During the subacute phase, edema may decrease, and lesions may become more focal, localizing to the anterior horn region. Several weeks to months after symptom onset, gray matter lesions may resolve, whereas ventral nerve root enhancement, which is typically absent early, may become apparent [2,4,6,7]. MRI obtained outside the optimal diagnostic window may therefore fail to show characteristic gray matter lesions. These temporal changes can complicate the diagnosis, particularly when later imaging shows isolated nerve root enhancement without gray matter lesions, mimicking the radiologic appearance of GBS [2,4,6,7]. Notably, MRI abnormalities in AFM may not always correspond precisely to the clinical distribution of weakness [6]. In our patient, ventral nerve root enhancement was bilateral, whereas weakness remained confined to the left leg. 

Current AFM diagnostic criteria are largely based on acute-phase findings and require evidence of spinal gray matter involvement on MRI [2,5]. Beyond the acute stage, no validated diagnostic criteria are available for AFM. Key diagnostic findings, including MRI abnormalities, CSF pleocytosis, and viral detection, may diminish or become undetectable over time, making delayed presentations particularly difficult to diagnose [2,4]. Enterovirus detection is most likely early in the clinical course [2]. Therefore, the negative enterovirus results in our patient should be interpreted cautiously because testing was performed approximately one month after symptom onset. The lack of diagnostic criteria beyond the acute stage may contribute to under-recognition or misclassification of AFM and may lead to underestimation of its true incidence. Although some patients present in the subacute stage, when characteristic findings may no longer be evident, recognition remains important for rehabilitation planning, prognosis, and family counseling. In our case, isolated left leg weakness initially prompted evaluation for orthopedic causes. Spinal MRI obtained in the subacute stage reasonably suggested GBS. At that time, the diagnosis remained uncertain because characteristic gray matter lesions were absent. During longitudinal follow-up, however, persistent unilateral weakness and progressive neurogenic atrophy increasingly supported AFM as the more likely diagnosis, underscoring the importance of interpreting MRI findings in the context of disease timing. 

## Conclusions

AFM should be considered in children presenting with AFP. Clinicians should be cautious not to rely solely on strict acute-phase criteria, as patients presenting in later stages may no longer exhibit typical findings. Diagnostic criteria applicable beyond the acute stage may help improve diagnostic accuracy and guide appropriate long-term management.
